# Supplementary material for: A Critical View of the Application of the APEX Software (Aqueous Photochemistry of Environmentally-Occurring Xenobiotics) to Predict Photoreaction Kinetics in Surface Freshwaters
Source: Molecules. 2019 Dec 18;25(1):9. doi: 10.3390/molecules25010009 (PMC7017383; doi:10.3390/molecules25010009)
Supplement: Supplementary file 1 [file molecules-25-00009-s001.zip › 3.2.4_gcc-4.4.0.zip!/3.2.4_gcc-4.4.0/3.2.4_gcc-4.4.0/doc/octave/html/interpreter/Colors.html]

Colors - Untitled


Next: Line Styles,
Previous: Managing Default Properties,
Up: Advanced Plotting


---

#### 15.2.4 Colors

Colors may be specified as RGB triplets with values ranging from zero to
one, or by name. Recognized color names include `"blue"`,
`"black"`, `"cyan"`, `"green"`, `"magenta"`,
`"red"`, `"white"`, and `"yellow"`.
